# Can flaxseed supplementation affect circulating adipokines in adults? An updated systematic review and meta-analysis of randomized controlled trials

**DOI:** 10.3389/fnut.2023.1179089

**Published:** 2023-09-07

**Authors:** Shaghayegh Abbasi, Kiana Karimi, Amir Hossein Moridpour, Vali Musazadeh, Amir Hossein Faghfouri, Hannane Jozi

**Affiliations:** ^1^Department of Pharmacy, Shahid Sadoughi University of Medical Sciences, Yazd, Iran; ^2^Department of Nutrition, Isfahan University of Medical Sciences, Isfahan, Iran; ^3^Student Research Committee, Tabriz University of Medical Sciences, Tabriz, Iran; ^4^School of Nutrition and Food Sciences, Tabriz University of Medical Sciences, Tabriz, Iran; ^5^Maternal and Childhood Obesity Research Center, Urmia University of Medical Sciences, Urmia, Iran

**Keywords:** flaxseed, adiponectin, leptin, meta-analysis, systematic review

## Abstract

**Introduction:**

The findings of randomized controlled trials (RCTs) regarding the effect of flaxseed on adipokine concentrations are conflicting. Therefore, the present meta-analysis was conducted to provide definite and conclusive results.

**Methods:**

Systematically, Scopus, Embase, PubMed, Web of Science databases, and Google Scholar were searched for relevant literature published up to December 2022. Based on random-effect models, standard mean differences (SMDs) were calculated for net changes in adipokine concentrations.

**Results:**

Overall, 13 RCTs (15 arms) were eligible to be included. The results indicated that leptin was significantly reduced after the intervention with flaxseed supplement (SMD = −0.69, 95% CI: −1.37, −0.01; *p* = 0.048; *I*^2^ = 92.0%, *p* < 0.001). In addition, flaxseed supplements had no considerable effect on plasma adiponectin (SMD = 0.52, 95% CI: −0.20, 1.25, *p* = 0.159; *I*^2^ = 92.0%, *p* < 0.001).

**Discussion:**

Flaxseed significantly improves leptin but does not affect adiponectin concentrations. Additional future well-designed trials are required to further assess the potential benefits of flaxseed on adipokines in humans.

## 1. Introduction

The most common circulating hormone secreted by adipocytes is adiponectin. Adiponectin regulates many metabolic pathways, including fatty acid modulation and glucose oxidation (1). The high-molecular weight (HMW) of adiponectin is also considered a risk factor for metabolic syndrome (MetS) (2, 3). Previous studies have demonstrated that adiponectin levels are lowered in people with type 2 diabetes (T2DM), MetS, and cardiovascular disease (CVD) (4). High plasma levels of adiponectin have also been associated with a lower risk of myocardial infarction in men (5). Leptin is a hormone that regulates energy intake and consumption. It may also have an effect on the pathways that regulate glycolytic enzyme activity, glucose uptake, and the production of inflammatory cytokines (6–8).

Flaxseed (*Linum usitatissimum*), which is an oil seed or grain, has been suggested as a possible functional food since it contains bioactive components (9). Alpha-linolenic acid (ALA), which makes up ~55% of the total fatty acid content, is present in high amounts. Lignans, a group of phytoestrogens, are also present. There is also dietary fiber that makes up 28% of the weight, and up to one-third is soluble fiber (10). These characteristics suggest that flaxseed may have anti-inflammatory effects and clinical intervention trials have been conducted to ascertain whether flaxseed and flaxseed-derived products (flaxseed oil, whole flaxseed, or lignans) are effective in reducing a variety of cardiovascular risk factors, especially inflammatory indicators such as C-reactive protein (11–14).

In addition to the potential anti-inflammatory capabilities of flaxseed, adiponectin expression has been found to be induced by several flaxseed components in preclinical animal models (15). Additionally, flaxseed oil increased the expression of hepatic adiponectin receptors and circulating adiponectin (16). Other investigations have reached the conclusion that variations in leptin expression may contribute to the possible cardioprotective benefits of flaxseed supplementation (17). According to experimental research, ALA can bind to peroxisome proliferator-activated receptor gamma (PPARγ), which can enhance adiponectin expression and levels in the blood (18, 19). Other clinical trials have reported that ALA enhances adiponectin; in fact, ALA and adiponectin production was found to have a dose–response relationship (20). However, randomized controlled trials (RCTs) have shown conflicting outcomes. The effects of flaxseed on adiponectin and leptin levels were evaluated in a previous meta-analysis published in 2020 (21); however, several trials did not fully measure changes in their concentration. We conducted an additional study on the effects of flaxseed on leptin and adiponectin levels in adults as a result of the contradictory findings of the previous studies and the lack of a comprehensive meta-analysis. To determine the effect of flaxseed supplementation and flaxseed-derived products on adiponectin and leptin levels, the present study performed a comprehensive systematic review and meta-analysis of all relevant RCTs in adults.

## 2. Methods

This systematic review and meta-analysis was carried out and reported under the Preferred Reporting Items of Systematic Reviews and Meta-Analysis (PRISMA) statement guidelines (22).

### 2.1. Search strategy

We searched international databases, including Scopus, Embase, PubMed, Web of Science databases, and Google Scholar, from inception to December 2022, using the following keywords: (flax OR flaxseed OR linseed OR lignan OR whole flaxseed OR ground flaxseed OR flaxseed oil OR *L. usitatissimum*) **AND** (adiponectin OR adipocytokines OR leptin). The search strategy is presented in Supplementary Table S1. The search process was conducted by two researchers (VM and AHM). In addition, reference lists were searched from included studies.

### 2.2. Eligibility criteria

Retrieved studies were included in our meta-analysis if they met the following evidence-based PICOS criteria: (1) Patients: adult individuals >18 years old; (2) Intervention: flaxseed supplementation; (3) Control: placebo or control; (4) Outcomes: sufficient data for extraction regarding adiponectin and leptin levels; and (5) Study design: RCTs. *In vitro, in vivo*, and *ex vivo* studies, observational studies, quasi-experimental studies, and animal studies were excluded from this meta-analysis. Only articles in English were included in the study.

### 2.3. Data extraction

Two independent researchers (SA and VM) screened and extracted data from each qualified trial. First author's name, publication year, study location, study design, sample size in each group, dose and type of flaxseed, duration of intervention, average age, gender and baseline body mass index (BMI) of subjects, and mean and standard deviation (SD) of adipokines in both groups at baseline and at the end of the study and their changes from baseline were extracted from the selected RCTs. Any disagreement about the choice of studies was settled by consensus (AHF).

### 2.4. Quality assessment and assessment of the meta-evidence

The methodological quality assessments of each included study were performed independently by at least two researchers using the Cochrane Collaboration risk of bias tool, in which domains were judged as “low-risk, high-risk, or unclear” (23). The credibility of RCTs was evaluated using the Grading of Recommendations, Assessment, and Evaluation (GRADE) approach, which consisted of five factors as follows: risk of bias, consistency of results, directness, precision, and potential for publication bias. The evidence is categorized into four categories, namely high, moderate, low, or very low.

### 2.5. Statistical analysis

The STATA program (version 16) was used to conduct the statistical analysis (Stata Corp, College Station, TX). To assess the effect size for adipokines, SD and mean differences were determined for the two groups. Furthermore, a random-effects model was used to estimate standardized mean differences (SMDs) with 95% confidence intervals (CIs) (24). When standard error (SE) or confidence interval (CI) was reported, they were also transformed into SD. Heterogeneity between studies was assessed using *I*^2^ and the *p*-value of Cochran's *Q*-test. We performed a subgroup analysis according to baseline BMI (< 30, ≥30), study quality (high and low), intervention duration (< 12 and ≥12 weeks), type of flaxseed (whole flaxseed and flaxseed oil), sample size (≤ 40 and >40), the health condition [T2DM, polycystic ovary syndrome (PCOS), obesity, and others], gender (men, women, and both), and average age (< 50 and ≥50 years) to identify potential sources of heterogeneity. We also performed a sensitivity analysis to determine the effect of removing one particular study from the overall SMDs. Begg's adjusted rank correlation and Egger's regression asymmetry test were applied to examine the results of the small study effect (25, 26). Publication bias was assessed by visual inspection of funnel plots. If there was evidence of publication bias, the “trim and fill” method was carried out. All statistical tests were two-sided, and a *p*-value of < 0.05 was considered statistically significant.

## 3. Results

### 3.1. Flow and characteristics of included studies

**Figure 1:**
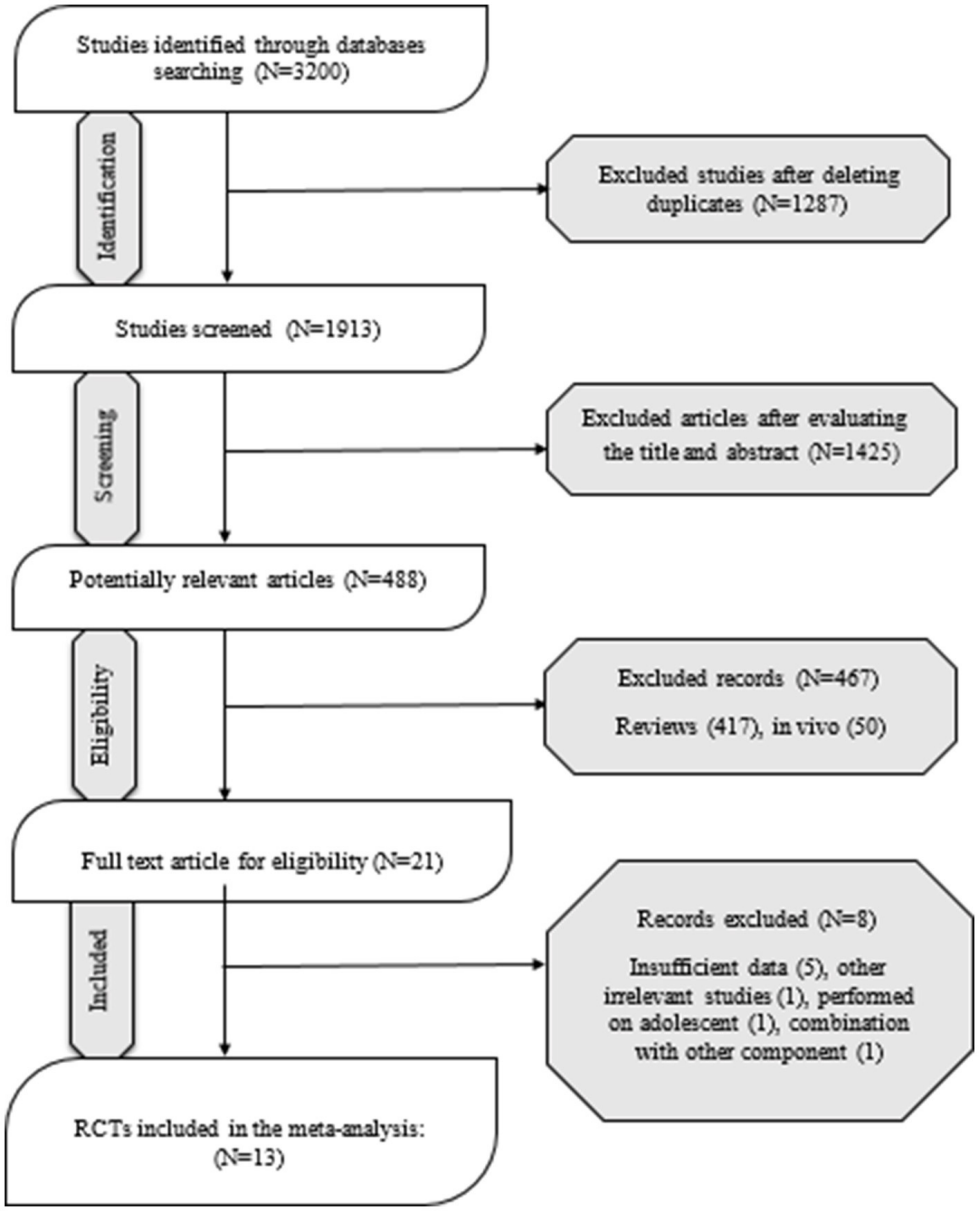
PRISMA flowchart diagram.

A total of 3,200 studies were identified in the databases, and 1,287 duplicates were excluded. In total, 1,425 studies were evaluated based on the title and abstract, and 467 were deemed irrelevant. There were 21 studies that went through a full-text evaluation, and 8 were omitted. Finally, 13 studies were included in the analysis. Figure 1 shows the selection process of the study. Studies were conducted in Iran (27, 28), China (29), Canada (30), USA (31–34), Greece (35, 36), and Brazil (37–39). The range of intervention periods varied from 2 to 12 weeks. Whole flaxseed (27–30, 38, 39) and ground flaxseed (33) were used in four RCTs with doses from 13,000 to 60,000 mg/day. In the other studies, flaxseed oil (30–32, 34–37) was used, with doses of 3,500 to 14,200 mg/day. In this study, different patient populations were examined in eligible RCTs. Included subjects were patients with obesity (27, 29, 31, 38, 39), dyslipidemia (35), T2DM (30, 37), pre-diabetes (33), and PCOS (28, 32, 34), and healthy people (36). Detailed characteristics of the included studies are summarized in Table 1.

**Table 1:** Study characteristics of included studies.

**Author, year**	**Design**	**Participants, *n***	**Health condition**	**Age, year**	**Intervention**		**Baseline adiponectin**	**Baseline leptin**	**Duration (week)**
					**Treatment group**	**Control group**			
Paschos et al. (35)	RA/SB/parallel	M: 35 Int: 18, Con: 17	Dyslipidemia	Int: 49, Con: 54	15 ml/day flaxseed oil	Safflower oil	Int: 5.97 μg/ml, Con: 5.98 μg/ml	–	12
Nelson et al. (31)	RA/parallel	M/F: 57 Int: 30, Con: 27	Overweight and obese	Int: 38.8, Con: 38.15	11,000 mg/day flaxseed oil (capsule)	Normal diet	Int: 10.12 μg/ml, Con: 7.93 μg/ml	–	8
Faintuch et al. (38)	RA/DB/crossover	M/F: 24 Int: 14, Con: 10	Obese	40.8	30,000 mg/day flaxseed flour	Manioc flour	–	Int: 27.3 ng/ml, Con: 27 ng/ml	2
Taylor et al. (30)	RA/parallel	M/F: 22 Int: 13, Con: 9 • M/F: 21 Int: 12, Con: 9	T2DM	52.4	32,000 mg/day milled flaxseed • 13,000 mg/day flaxseed oil	Placebo	Int: 10.5 μg/ml, Con: 9.8 μg/ml • Int: 6.9 μg/ml, Con: 9.8 μg/ml	Int: 10 ng/ml, Con: 25 ng/ml • Int: 10.3 ng/ml, Con: 25 ng/ml	12
Faintuch et al. (39)	RA/SB/parallel	M/F: 28 Int: 10, Con: 18	Obese	Int: 47.8, Con: 50.7	60,000 mg/day flaxseed powder	Cassava powder	–	Int: 44.4 ng/ml, Con: 27.6 ng/ml	12
Vargas et al. (32)	RA/DB/parallel	F: 34 Int: 17, Con: 17	PCOS	Int: 29.4, Con: 28.9	3,500 mg/day flaxseed oil (capsule)	Soybean oil	Int: 8 ng/ml, Con: 6.5 ng/ml	Int: 27.1 ng/ml, Con: 28.1 ng/ml	6
Kontogianni et al. (36)	RA/crossover	M/F: 37 Int: 19, Con: 18	Healthy	25.6	13,800 mg/day flaxseed oil	Olive oil	Int: 6.2 mg/L • Con: 6.5 mg/L	–	6
Hutchins et al. (33)	RA/crossover	M/F: 25 Int: 13, Con: 12 •M/F: 25 Int: 13, Con: 12	Pre-diabetes	58.6	13,000 mg/day ground flaxseed • 26,000 mg/day ground flaxseed	Placebo	Int: 8.4 μg/ml, Con: 9.4 μg/ml • Int: 9.3 μg/ml, Con: 9.4 μg/ml	–	12
Gomes et al. (37)	RA/DB/parallel	M/F: 20 Int: 10, Con: 10	T2DM	Int: 47, Con: 50.1	6,000 mg/day linseed oil (capsule)	Placebo	Int: 10.61 μg/ml, Con: 12.04 μg/ml	–	8
Karakas et al. (34)	RA/DB/parallel	F: 34 Int: 17, Con: 17	PCOS	Int: 29.4, Con: 28.9	3,500 mg/day flaxseed oil (capsule)	soybean oil	Int: 8 ng/ml, Con: 6.5 ng/ml	Int: 27.1 ng/ml, Con: 28.1 ng/ml	6
Haidari et al. (28)	RA/parallel	F: 41 Int: 21, Con: 20	PCOS	Int: 27.21, Con: 26.13	30,000 mg/day brown milled flaxseed powder + lifestyle modification	Lifestyle modification	Int: 13.04 mg/ml, Con: 14.56 mg/ml	Int: 70.18 ng/ml, Con: 64.64 ng/ml	12
Kuang et al. (29)	RA/DB/parallel	M/F: 51 Int: 27, Con: 24	Overweight and obese	Int: 22.74, Con: 21.79	13,000 mg/day flaxseed meal (Biscuits)	Control	Int: 21.89 μg/ml, Con: 25.52 μg/ml	Int: 12.25 ng/ml, Con: 12.19 ng/ml	8
Ahmadniay motlagh et al. (27)	RA/DB/parallel	F: 52 Int: 29, Con: 23	Overweight and obese	Int: 38.28, Con: 41.74	30,000 mg/day brown milled flaxseed powder	Raw milled rice	Int: 12.11 ng/ml, Con: 12.48 ng/ml	Int: 53.76 ng/ml, Con: 51.48 ng/ml	12

### 3.2. Risk of bias assessment and quality of evidence

Random allocation of participants was mentioned in all included trials. Most of the included studies had a low/unclear risk of allocation concealment and reporting bias. In addition, most studies showed a high risk of other sources of bias and detection bias. Out of the 13 RCTs in the current study, five were of high quality (27–29, 32, 34), six were of moderate quality (33, 35–39), and two were of low quality (30, 31). Detailed information regarding the quality of the included RCTs based on the Cochrane risk of bias assessment is shown in Table 2. GRADE quality of evidence was high for leptin and moderate for adiponectin (Table 3).

**Table 2:** Results of risk of bias assessment for randomized clinical trials included in the current meta-analysis on the effects of flaxseed supplementation on adipokines in adults.

**Study**	**Random sequence generation**	**Allocation concealment**	**Reporting bias**	**Other sources of bias**	**Performance bias**	**Detection bias**	**Attrition bias**
Paschos et al. (35)	L	L	L	H	L	H	L
Nelson et al. (31)	L	U	L	L	H	H	H
Faintuch et al. (38)	L	U	L	H	L	L	L
Taylor et al. (30)	L	U	L	H	U	U	H
Faintuch et al. (39)	L	U	L	H	L	H	L
Vargas et al. (32)	L	L	L	H	L	L	L
Kontogianni et al. (36)	L	L	L	H	U	U	L
Hutchins et al. (33)	L	L	L	H	U	U	L
Gomes et al. (37)	L	U	L	H	L	L	H
Karakas et al. (34)	L	L	L	L	L	L	L
Haidari et al. (28)	L	L	L	L	U	U	L
Kuang et al. (29)	L	L	L	H	L	L	L
Ahmadniay motlagh et al. (27)	L	L	L	L	L	L	L

Each study was assessed for risk of bias using the Cochrane Risk of Bias Assessment tool. Domains of assessment were included random sequence generation, allocation concealment, reporting bias, performance bias, detection bias, attrition bias, and other sources of bias. Each domain was scored as “high risk” if it contained methodological flaws that may have affected the results, “low risk” if the flaw was deemed inconsequential, and “unclear risk” if information was insufficient to determine. If a study got “low risk” for all domains, it is considered a high-quality study with low total risk of bias.

**Table 3:** GRADE profile of flaxseed supplementation on plasma adipokines.

**Adipokines**	**Summary of findings**		**Quality of evidence assessment (GRADE)**					
	**No. of patients (trials)**	SMD^*^(95% CI)	**Risk of bias^a^**	**Inconsistency^b^**	**Indirectness^c^**	**Imprecision**	**Publication bias^e^**	**Quality of evidence^f^**
Adiponectin	420 (11)	0.52 (−0.20, 1.25)	Not serious	Not serious	Not serious	Serious^d^	Not serious	Moderate
Leptin	288 (8)	−0.69 (−1.37, −0.01)	Not serious	Not serious	Not serious	Not serious	Not serious	High

^*^Presented as standard mean difference (SMD) all outcomes.

^a^Risk of bias based on the Cochrane risk of bias tool. This tool assesses selection bias, performance bias, detection bias, attrition bias, and reporting bias. Five of the eight included studies had incomplete outcome data (attrition bias). Half of the included studies had performance bias.

^b^Downgraded if there was a substantial unexplained heterogeneity (I^2^ > 50%, p < 0.10) that was unexplained by meta-regression or subgroup analyses.

^c^Downgraded if there were factors present relating to the participants, interventions, or outcomes that limited the generalizability of the results.

^d^There is no evidence of significant effects of flaxseed supplementation on adiponectin (95% CI including 0).

^e^Downgraded if there was evidence of publication bias using a funnel plot that affected overall results detected by trim and fill analysis.

^f^Since all included studies were randomized controlled trials, the certainty of the evidence was graded as high for all outcomes by default and then downgraded based on prespecified criteria. Quality was graded as high, moderate, low, and very low.

### 3.3. Flaxseed on adiponectin concentrations

**Figure 2:**
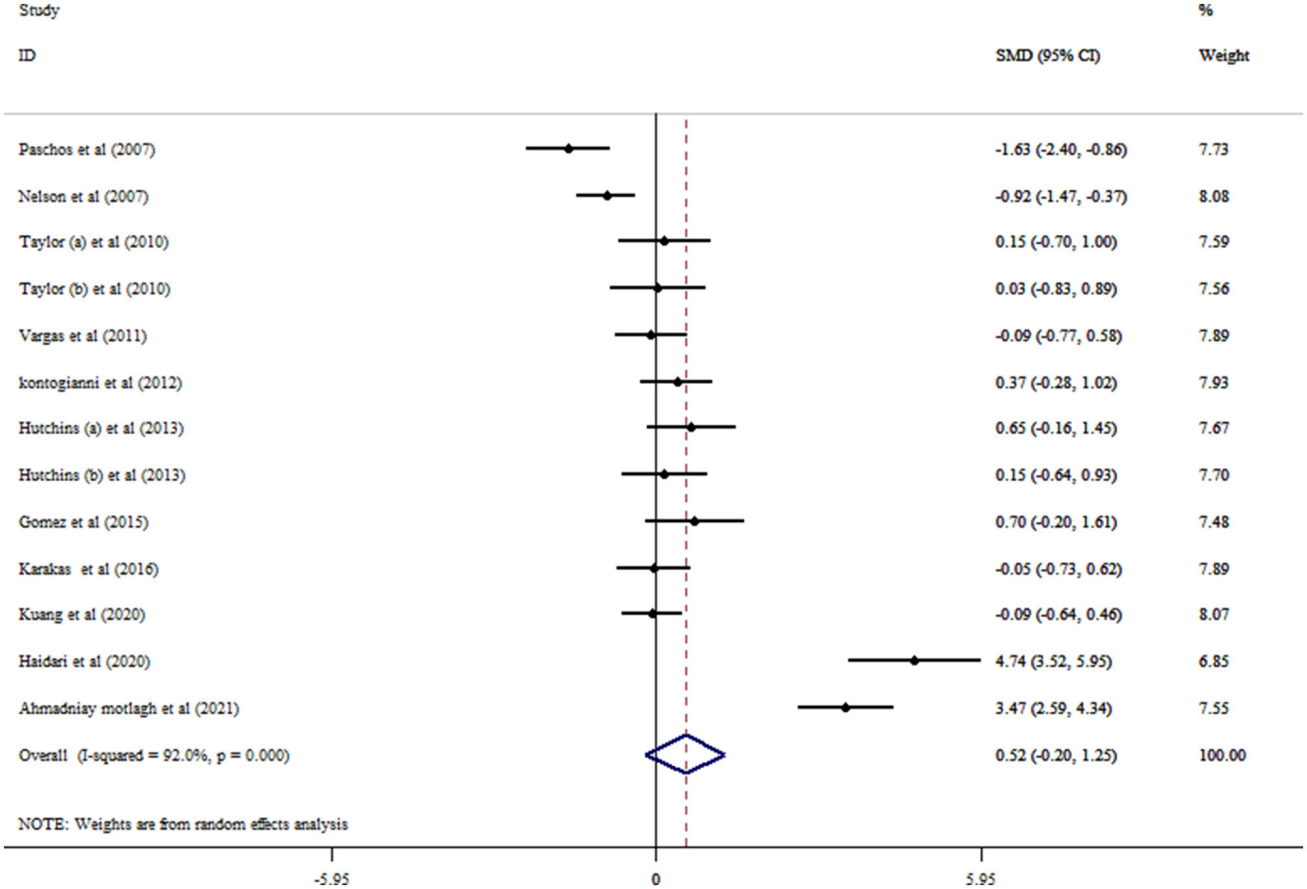
Forest plot detailing mean difference and 95% confidence intervals (CIs) and the effects of flaxseed supplementation on adiponectin levels.

**Figure 3:**
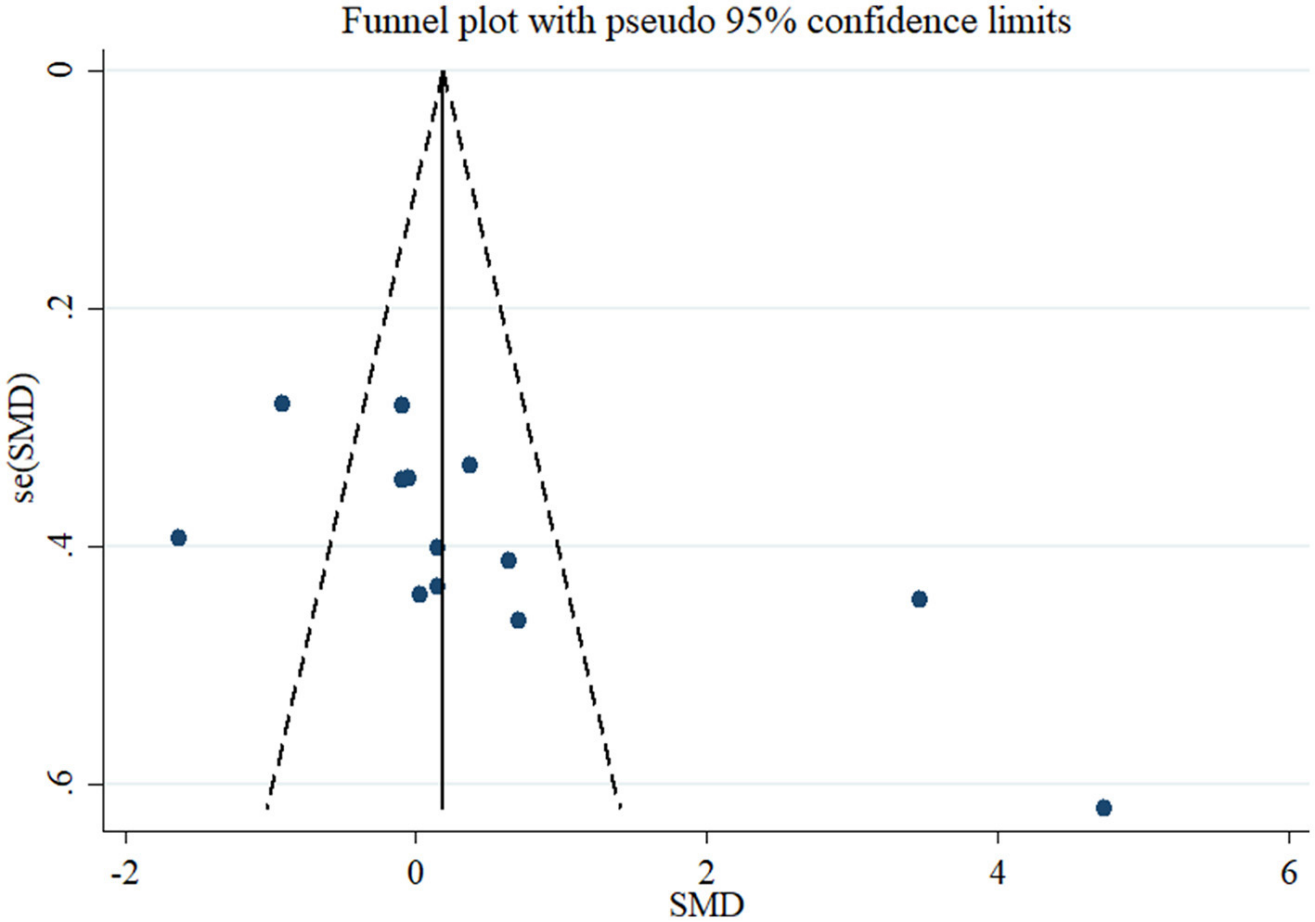
Funnel plot displaying publication bias in the studies reporting the effects of flaxseed supplementation on adiponectin levels.

Based on the result of 11 RCTs comprising 13 treatment arms, flaxseed could not significantly affect circulating adiponectin in adults (SMD = 0.52, 95% CI: −0.20, 1.25, *p* = 0.159; Figure 2). The results were heterogeneous (*I*^2^ = 92.0%, *p* < 0.001), and the sensitivity analysis results revealed no significant change following the removal of each study. Subgroup analysis indicated significant effects on adiponectin in RCTs administered with whole flaxseed (Table 4). Egger's and Begg's tests showed significant small-study effects (*p* < 0.05). The trim and fill method was performed (without imputed study) following the uneven distribution of the funnel plot (Figure 3).

**Table 4:** Subgroup analyses for the effects of flaxseed supplementation plasma adipokines.

	**NO**	**SMD (95% CI)^a^**	***p*-within^b^**	***I*^2^ (%)^c^**	***p*-heterogeneity^d^**
**Flaxseed supplementation on adiponectin**					
Overall	13	0.52 (−0.20, 1.25)	0.159	92.0	< 0.001
**Age (year)**					
< 50	9	0.67 (−0.36, 1.69)	0.203	94.6	< 0.001
≥50	4	0.25 (−0.16, 0.66)	0.232	0.0	0.731
**Gender**					
Men	1	−1.63 (−2.40, −0.86)	< 0.001	0.0	< 0.001
Women	4	1.97 (−0.21, 4.15)	0.076	96.5	< 0.001
Both	8	0.08 (−0.32, 0.48)	0.698	59.4	0.016
**Intervention duration (week)**					
< 12	6	−0.06 (−0.50, 0.37)	0.774	63.3	0.018
≥12	7	1.05 (−0.42, 2.51)	0.161	95.1	< 0.001
**Intervention type**					
Whole flaxseed	6	1.46 (0.06, 2.86)	0.040	94.5	< 0.001
Flaxseed oil	7	−0.25 (−0.81, 0.32)	0.393	77.4	< 0.001
**Study population**					
Overweight and obese	5	0.62 (−0.68, 1.93)	0.351	94.5	< 0.001
PCOS	3	1.47 (−0.91, 3.85)	0.227	96.2	< 0.001
T2DM	3	0.28 (−0.22, 0.78)	0.275	0.0	0.535
Other diseases	2	−0.62 (−2.57, 1.34)	0.537	93.4	< 0.001
**Sample size**					
≤ 40	9	0.02 (−0.42, 0.46)	0.927	65.9	0.003
>40	4	1.75 (−0.54, 4.04)	0.135	97.5	< 0.
**BMI**					
≤ 30	5	0.76 (−0.75, 2.26)	0.325	94.8	< 0.001
>30	8	0.40 (−0.44, 1.24)	0.352	90.4	< 0.001
**Study quality**					
Low	9	0.41 (−0.48, 1.30)	0.365	91.3	< 0.001
High	4	0.78 (−0.66, 2.22)	0.289	94.5	< 0.001
**Flaxseed supplementation on leptin**					
Overall	9	−0.69 (−1.37, −0.01)	0.048	86.4	< 0.001
**Age (year)**					
< 50	7	−0.84 (−1.68, −0.01)	0.047	89.0	< 0.001
≥50	2	−0.16 (−0.93, 0.61)	0.686	36.4	0.210
**Gender**					
Women	4	−1.37 (−2.75, 0.01)	0.051	93.1	< 0.001
Both	5	−0.21 (−0.76, 0.33)	0.442	56.9	0.055
**Intervention duration (week)**					
< 12	4	−0.32 (−0.81, 0.17)	0.198	52.0	0.100
≥12	5	−1.06 (−2.37, 0.25)	0.112	91.6	< 0.001
**Intervention type**					
Whole flaxseed	6	−1.03 (−2.02, −0.05)	0.040	89.6	< 0.001
Flaxseed oil	3	−0.09 (−0.50, 0.33)	0.686	0.0	0.690
**Study population**					
Overweight and obese	4	−0.48 (−1.20, 0.23)	0.181	74.9	0.007
PCOS	3	−1.47 (−3.57, 0.63)	0.170	95.3	< 0.001
T2DM	2	−0.16 (−0.93, 0.61)	0.686	36.4	0.210
**Sample size**					
≤ 40	6	−0.06 (−0.39, 0.26)	0.693	0.0	0.727
>40	3	−2.02 (−3.55, −0.50)	0.009	92.6	< 0.001
**BMI**					
≤ 30	2	−2.53 (−5.73, 0.66)	0.120	96.1	< 0.001
>30	7	−0.25 (−0.68, 0.18)	0.257	55.5	0.036
**Study quality**					
Low	4	−0.65 (−1.16, −0.14)	0.013	61.8	0.049
High	5	−0.78 (−2.23, 0.66)	0.289	92.1	< 0.001

^a^Obtained from the Random-effects model.

^b^Refers to the mean (95% CI).

^c^Inconsistency, percentage of variation across studies due to heterogeneity.

^d^Obtained from the Q-test.

SMD, standard mean differences; CI, confidence interval; NR, not reported; NAFLD, non-alcoholic fatty liver disease.

### 3.4. Flaxseed on leptin concentrations

**Figure 4:**
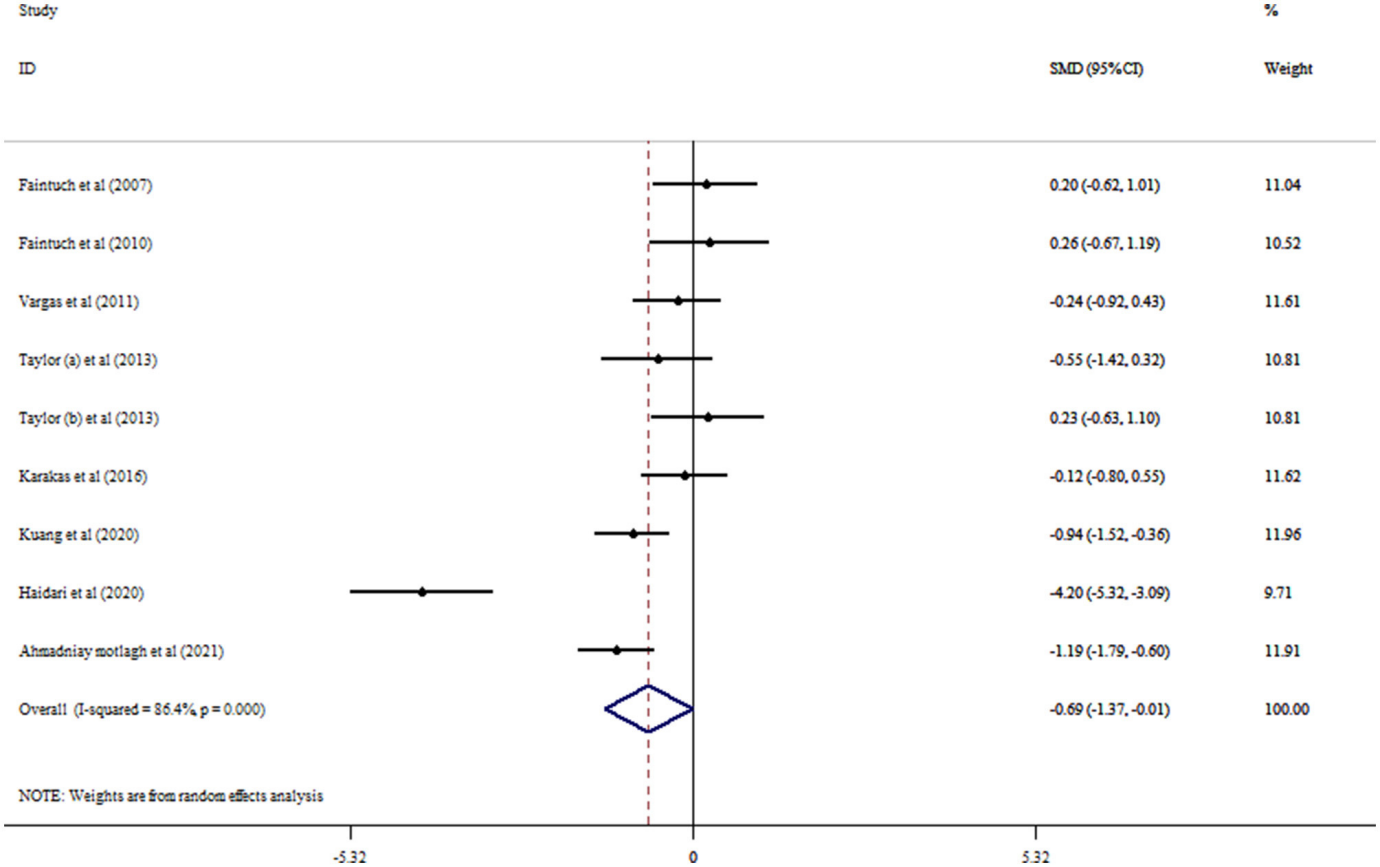
Forest plot detailing mean difference and 95% confidence intervals (CIs) and the effects of flaxseed supplementation on leptin levels.

Eight RCTs with nine arms investigated the effect of flaxseed supplementation on leptin levels. The results indicated a significant reducing effect of flaxseed supplementation on leptin levels (SMD = −0.69, 95% CI: −1.37, −0.01; *p* = 0.048) with between-study heterogeneity (*I*^2^ = 86.4%, *p* < 0.001; Figure 4). Moreover, the overall effects of flaxseed on leptin were changed to not significantly impact by excluding studies using a one-study removal analysis (27–29, 32, 34). Whole flaxseed supplementation among RCTs with a sample size of >40 participants and age < 50 years contributed to a robust reduction in leptin concentrations (Table 4). Begg's tests showed no significant publication bias (*p* = 0.754).

## 4. Discussion

The results of our pooled analysis showed that flaxseed supplementation, despite its non-significant effect on adiponectin, caused a significant decrease in circulating leptin. However, subgroup analysis showed that flaxseed had no significant effect on leptin levels in high-quality studies, and only low-quality studies showed ameliorating effects of flaxseed on leptin. Consequently, the interpretation of this result should be accompanied by caution, and studies with appropriate designs and a low risk of bias are needed to confirm our results on leptin. In addition, the examination of other subgroups showed that flaxseed significantly increases adiponectin in the form of whole flaxseed. Regarding leptin, the whole flaxseed caused a significant decrease in people of < 50 years of age and in studies with a sample size of over 40. The results of the previous meta-analysis study in 2019 by Jalili et al. (21) showed that flaxseed supplementation has no significant effect on leptin and adiponectin levels and also on studied subgroups. However, the abovementioned study suggested that additional clinical trial studies should also be conducted for a definitive conclusion. Our study added four more clinical trials (27–29, 34) than Jalili et al.'s study of the pooled analysis which yielded different results in some aspects. In addition, the subgroup analysis of Jalili et al.'s study was only limited to the duration of the supplementation, the study population (healthy or unhealthy), and the type of intervention. However, our study added demographic variables (age and gender), sample size, body mass index, and study quality to subgroup analysis and examined the studied population more comprehensively than the aforementioned study in order to obtain generalizable results. In addition, unlike the abovementioned study, the quality of the obtained evidence in our investigation was checked with the GRADE tool.

In both studied biomarkers, whole flaxseed compared with flaxseed oil led to a significant improvement in leptin and adiponectin levels. Unlike flaxseed oil, which contains omega-3 fatty acids, especially polyunsaturated fatty acids (PUFAs), whole flaxseed contains PUFAs, soluble and insoluble fibers, proteins, various antioxidants, and phytoestrogenic lignans (40) that explain more improving effects on adipokines.

Studies have pointed out that the circulating levels of adiponectin and leptin are higher in women than in men (41, 42). Due to the existence of only one low-quality study (34) in the subgroup of men, the significant reduction of adiponectin in this subgroup cannot be a generalizable and valid result. No significant results have been reported for other gender subgroups either in leptin or adiponectin. However, it is suggested that future studies separate the effect of flaxseed supplements in men and women, in order to report a more accurate result. In terms of mean age, only one low-quality study (30) with two investigated arms included a < 50 years of age subgroup in the leptin-pooled analysis. Therefore, a significant decrease in leptin in this subgroup is not highly worth noting. However, this finding can be a sign for future studies to clarify the effect of flaxseed on this age group.

The sample size is another important factor determining the true effect of flaxseed on leptin. Subgroup analysis showed that studies with a >40 sample size reported a significant decrease in leptin levels following flaxseed supplementation. As a general principle in epidemiological studies, large sample sizes lead to high power to show a true effect (43). However, a very high sample size can also lead to false conclusions (44).

The various compounds found in whole flaxseed lead to beneficial effects on circulating levels of leptin and adiponectin. Fatty acids through interaction with transcription factors such as PPARγ, CCAAT/enhancer-binding protein (C/EBP), and sterol regulatory element-binding transcription factor 1 (SREBPF1) can alter the expression of leptin and adiponectin (45). Moreover, the anti-inflammatory properties of omega-3 fatty acids contribute to the regulation of adipokine production (46). Studies have reported that inflammatory conditions can lead to the inhibition of adiponectin production from adipocytes (47). In addition, pro-inflammatory cytokines have stimulating effects on leptin production (48). The main fatty acid of flaxseed oil is ALA, which is a poor activator of PPARγ compared with arachidonic acid as the main activator of PPARγ among fatty acids (49). This could explain the difference in the results observed between whole flaxseed and flaxseed oil. Dietary fibers can regulate the levels of adipokines in various ways, such as through changing body composition (50) and modifying gut microbiota (51). Due to the similar structure of phytoestrogens and estrogen, these compounds can bind to estrogen receptors (ERs) with a high affinity toward ERβ than ERα (52), leading to the inhibition of adipocyte differentiation and lipid accumulation in an *in vivo* model (53). However, phytoestrogens can directly bind to and activate PPARγ (52). This cross-talk between PPARγ and ERs focused on future studies to elucidate the precise effect of phytoestrogens on obesity-related pathways. The beneficial effects of plant polyphenols and antioxidants on the balance between different adipokines have been investigated in some studies (54, 55).

There were some limitations worth noting in our study that are suggested to cover in future studies. First, due to the lack of sufficient studies, an accurate comparison between men and women was not possible. As it is known, the expression of estrogen receptors between the two genders has a different pattern (56), and this can be effective in the effect of flaxseed on the circulating levels of adipokines. Second, it seems that the effect of flaxseed on other adipokines, such as visfatin and resistin, should also be taken into consideration in order to obtain a more comprehensive conclusion. Third, there were limited studied populations; therefore, additional studies on other diseases especially inflammatory conditions seem necessary.

Our study also has some worth noting strengths. First, the present study tried to cover all the limitations of the previous meta-analysis. Second, due to the low risk of bias in the included studies and the appropriate design of the current meta-analysis, the quality of our obtained results was moderate for adiponectin and high for leptin. Third, our study was registered in PROSPERO (code: CRD42023399735).

## 5. Conclusion

Whole flaxseed is effective in improving the levels of adiponectin and leptin. Flaxseed oil cannot change circulating levels of adipokines. The quality of our obtained results is moderate for adiponectin and high for leptin.

## Data availability statement

The original contributions presented in the study are included in the article/Supplementary material, further inquiries can be directed to the corresponding authors.

## Author contributions

VM was responsible for designing and coordinating the study. VM, KK, SA, HJ, and AH were responsible for the statistical study and writing of the manuscript. AF was responsible for reviewing the manuscript. KK was responsible for the statistical work and for writing the manuscript. All authors were responsible for data collection, data analysis, and data interpretation of the manuscript, and approved the final manuscript.

## Supplementary material

Supplementary Table S1 The search strategy.
