# Supplementary material for: Can flaxseed supplementation affect circulating adipokines in adults? An updated systematic review and meta-analysis of randomized controlled trials
Source: Front Nutr. 2023 Sep 7;10:1179089. doi: 10.3389/fnut.2023.1179089 (PMC10513937; doi:10.3389/fnut.2023.1179089)
Supplement: Supplementary Table S1 — The search strategy. [file Data_Sheet_1.docx]

The search was conducted using the following search pattern:

[Flax [MeSH] OR flaxseed [Title/Abstract] OR flaxseed [Title/Abstract] OR linseed [tiab] OR lignan [Title/Abstract] OR “whole flaxseed” [tiab] OR “ground flaxseed” [Title/Abstract] OR “flaxseed oil” [tiab] OR “Linum usitatissimum” [Title/Abstract] AND Adipokines[Mesh] OR adipocytokines[Title/Abstract] OR Adiponectin [Mesh] OR ‘Leptin’[Mesh] AND "randomized controlled trial"[Publication Type] OR "controlled clinical trial"[Publication Type] OR "controlled clinical trial"[All Fields] OR randomized[Title/Abstract] OR randomized [Title/Abstract] OR placebo[Title/Abstract] OR "clinical trials as topic"[MeSH Terms] OR "cross-over studies"[MeSH Terms] OR "cross-over studies"[All Fields] OR "cross over studies"[All Fields] OR "Cross-over study"[All Fields] OR "Cross over study"[All Fields] OR "clinical trial"[Publication Type] NOT animals[All Fields].
